# Size Control of Magnetite Nanoparticles in Excess Ligands as a Function of Reaction Temperature and Time

**DOI:** 10.3390/molecules190811395

**Published:** 2014-08-04

**Authors:** Masafumi Nakaya, Ryo Nishida, Atsushi Muramatsu

**Affiliations:** 1 Institute of Multidisciplinary Research for Advanced Materials, Tohoku University, Sendai 980-8577, Japan; E-Mail: mura@tagen.tohoku.ac.jp; 2 School of Engineering, Tohoku University, Sendai 980-8579, Japan; E-Mail: ryo-24d@mail.tagen.tohoku.ac.jp

**Keywords:** Fe_3_O_4_ nanoparticle, solventless synthesis, size control

## Abstract

The novel synthesis of monodisperse magnetite Fe_3_O_4_ nanoparticles of varying sizes using a solventless synthetic method was developed. Iron salt was treated in excess oleylamine and oleic acid as ligands. The effect of the reaction temperature and time on the particle size was investigated and the particle sizes were easily tuned from 5.3 to 20.4 nm by changing the reaction temperature and time.

## 1. Introduction

Chemically synthesized superparamagnetic magnetite (Fe_3_O_4_) nanoparticles have many attractive features, including their ease of synthesis, narrow size distribution, chemical stability, and high dispersibility in various solvents [1,2,3,4,5,6,7,8]. In addition, Fe_3_O_4_ nanoparticles exhibit high biocompatibility [9,10,11,12]. Therefore, they have potential uses in the biomedical field, including as contrast agents for magnetic resonance imaging (MRI) [13,14,15,16,17,18], drug delivery systems [19,20,21], and hyperthermia agents [22,23,24]. In order for that potential to come to fruition, size control is crucial because their magnetic properties strongly depend on size. In general, ferri- and ferromagnetic properties appear in particles larger than 30 nm [6]. However, particles smaller than 30 nm are desired in the biomedical field to facilitate superparamagnetism. In addition, even in particles smaller than 30 nm, the magnetic properties strongly depend on the size [8,24]. Therefore, controlling the size of Fe_3_O_4_ nanoparticles is imperative in controlling the properties for various applications in the biomedical field.

In conventional synthetic procedures utilized to obtain superparamagnetic Fe_3_O_4_ nanoparticles with tunable sizes, seed-mediated growth is a very simple way to promote growth [3,4]. However, seed-mediated growth tends to broaden the size distribution because of a generation of nucleation in each step of the growth reaction. Hyeon *et al.* reported a synthetic method to control the size of iron oxide nanoparticles by changing the reaction temperature. The procedure can easily control the size of the nanoparticles and prepare the particles in large quantities, however, the crystal structure changed with the reaction temperature [7]. In order to control the crystal structure, it is better that the reaction be carried out under mild condition [25,26,27,28].

As one of the methods to prepare nanoparticles under mild conditions, we have examined the effect of complexation between metal ions and ligands [29,30]. Complexation is effective for suppressing nucleation and supplying metal ions slowly to growth. In order to form the complex efficiently, the metal salts are preferably dissolved and reacted in an excess amount of ligands without using conventional solvents, such as 1-octadecene, di-*n*-octylether, and benzyl ether. When using such organic solvents, the ratio of ligands to metal is low to form complexes efficiently. Here, we report a novel synthetic procedure for preparing Fe_3_O_4_ nanoparticles of various sizes by using a solventless synthesis, where oleylamine and oleic acid were used as ligands. In order to control particle sizes, the effect of reaction temperature and time was investigated. 

## 2. Results and Discussion

The relationship between the reaction conditions and particle sizes of the resulting nanoparticles is summarized in Table 1.

**Table 1:** Summary of the relationship between the reaction conditions and the particle sizes of the resulting nanoparticles.

Reaction temperature (°C)	Reaction time (h)		
	1	3	6
200		5.3 ± 0.6 nm	
250		8.2 ± 0.6 nm	
280	6.6 ± 1.0 nm	13.0 ± 0.9 nm	19.5 ± 1.7 nm
300		20.4 ± 2.2 nm	

**Figure 1:**
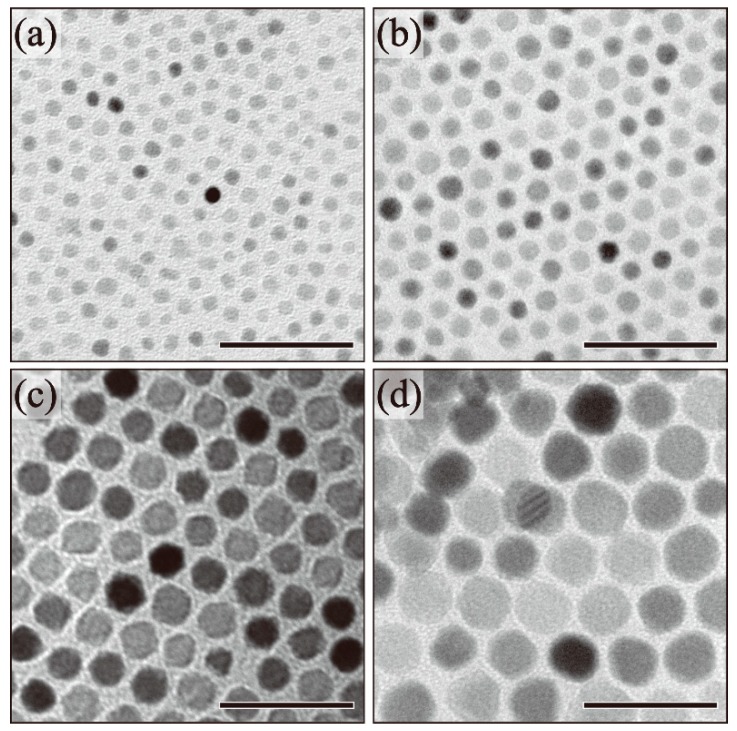
TEM images of (**a**) 5.3 ± 0.6 nm (200 °C); (**b**) 8.2 ± 0.6 nm (250 °C); (**c**) 13.0 ± 0.9 nm (280 °C); and (**d**) 20.4 ± 2.2 nm (300 °C) Fe_3_O_4_ nanoparticles. (Scale bar: 50 nm).

In order to understand the effect of reaction temperature on particle size, we obtained transmission electron microscope (TEM) images of the as-synthesized nanoparticles. The relationship between the reaction conditions and particle sizes of the resulting nanoparticles is summarized in Table 1. Figure 1 shows the TEM images of the nanoparticles as a function of reaction temperature. When the reaction temperature was 200 °C, the resulting nanoparticles exhibited spherical shapes with a particle size of 5.3 ± 0.6 nm, notably the minimum size observed in this study (Figure 1a). When the reaction temperature was increased to 250 °C, 280 °C, and 300 °C, the size of the spherical particles increased to 8.2 ± 0.6 nm, 13.0 ± 0.9 nm, and 20.4 ± 2.2 nm, respectively (Figure 1b–d). The particle size tended to increase with increased reaction temperatures. In our previous study, the iron ions in an excess amount of ligands formed complexes with oleylamine and oleic acid [29]. Through stable complex formation, nucleation was suppressed. In order to promote the decomposition of the stable iron complex, a higher reaction temperature was required. When 1,2-hexadecanediol was not added into the reaction solution, larger Wüstite phase particles were obtained [30]. 1,2-Hexadecanediol worked to suppress the growth of the particles larger than 20 nm by promotion of the nucleation number.

**Figure 2:**
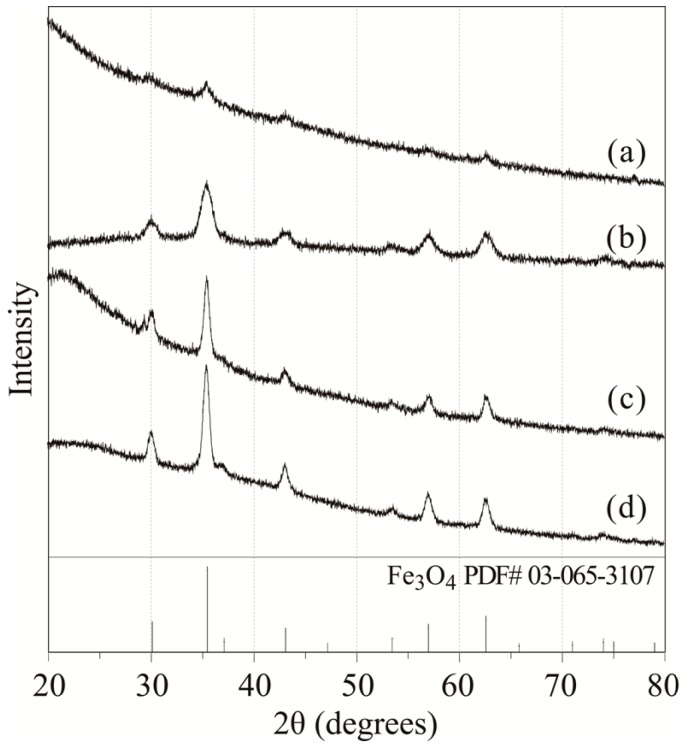
XRD patterns of (**a**) 5.3 ± 0.6 nm (200 °C); (**b**) 8.2 ± 0.6 nm (250 °C); (**c**) 13.0 ± 0.9 nm (280 °C); and (**d**) 20.4 ± 2.2 nm (300 °C) Fe_3_O_4_ nanoparticles.

The crystal structures of the as-synthesized nanoparticles were determined by X-ray diffraction (XRD). Figure 2 shows the XRD patterns of the resulting nanoparticles as a function of reaction temperature. When the reaction temperature was 200 °C, the observed peaks were small and broad (Figure 2a). Notably, 200 °C was too low of a temperature to improve the crystallinity, therefore, the peaks were not clear. However, when the reaction temperature was higher than 250 °C, the patterns corresponding to the Fe_3_O_4_ phase could be observed clearly (Figure 2b–d). The peak width narrowed slightly because the crystallinity and particle size improved with increasing temperatures [31,32]. The differences in the XRD patterns between Fe_3_O_4_ and γ-Fe_2_O_3_ phases are small. In order to determine the structure of the Fe_3_O_4_ phase, the coexistence of Fe^2+^ and Fe^3+^ in the particles must be demonstrated. 

**Figure 3:**
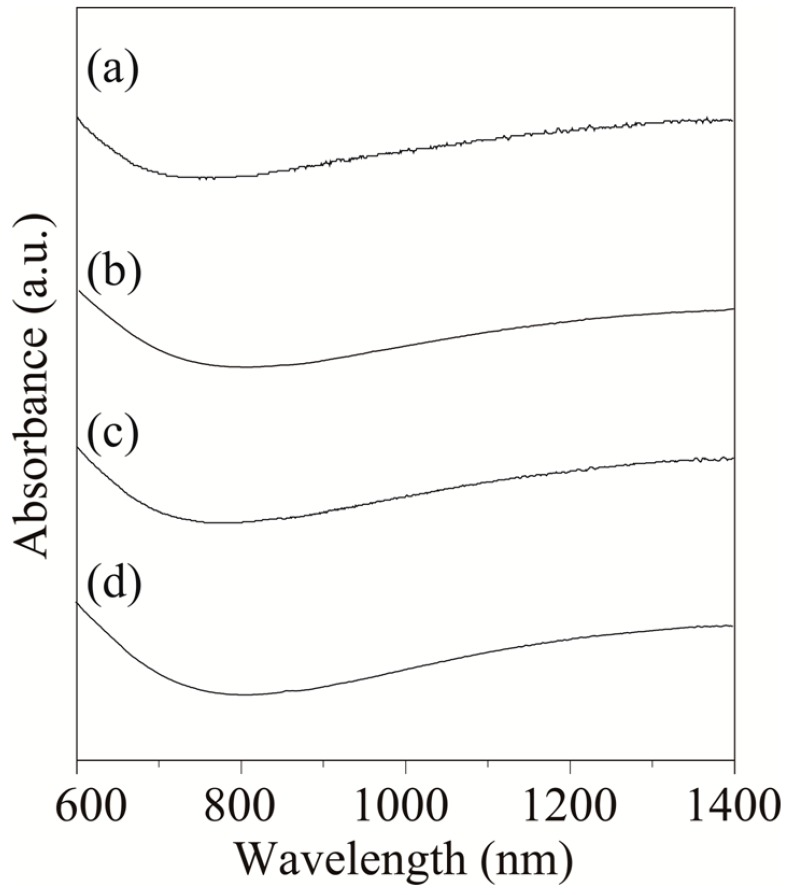
Vis-NIR spectra of (**a**) 5.3 ± 0.6 nm (200 °C); (**b**) 8.2 ± 0.6 nm (250 °C); (**c**) 13.0 ± 0.9 nm (280 °C); and (**d**) 20.4 ± 2.2 nm (300 °C) Fe_3_O_4_ nanoparticles.

In order to determine the valence of iron in the nanoparticles, the near-infrared absorption of the nanoparticles was measured. Adjacent Fe^2+^ and Fe^3+^ ions in the phase lead to electron transitions assigned to intervalence charge transfer (IVCT) transitions in the near-infrared (NIR) region [33,34]. However, the Fe^3+^ transition in the UV-Vis yields essentially the same spectrum in the presence or absence of nearby Fe^2+^ [34]. Therefore, the absorption spectra in the Vis-NIR region from 600 to 1,400 nm were used. Figure 3 shows the Vis-NIR spectra of the resulting nanoparticles in *n*-hexane (concentration: 0.005 wt%). The broad absorption in the NIR region demonstrated coexistence of Fe^3+^ and Fe^2+^ in the nanoparticles. These results support that the XRD patterns (Figure 2) shows magnetite, not maghemite.

**Figure 4:**
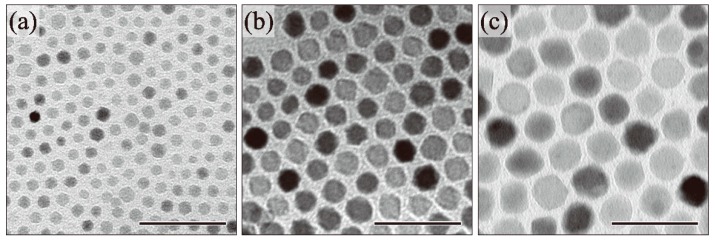
TEM images of (**a**) 6.6 ± 1.0 nm (1 h); (**b**) 13.0 ± 0.9 nm (3 h); and (**c**) 19.5 ± 1.7 nm (6 h) Fe_3_O_4_ nanoparticles. (Scale bar: 50 nm).

Next, we moved to an investigation of the effect of reaction time on particle size and structure. The relationship between the reaction conditions and particle sizes of the resulting nanoparticles is summarized in Table 1. Figure 4 shows the TEM images of the as-synthesized Fe_3_O_4_ nanoparticles as a function of reaction time. The reaction temperature was maintained at 280 °C for 1, 3, and 6 h. The mean diameter of the nanoparticles was 6.6 ± 1.0 nm, 13.0 ± 0.9 nm, and 19.5 ± 1.7 nm after 1 h, 3 h, and 6 h, respectively. Longer reaction times led to increase particle growth with narrow size distribution. However, when the reaction time was longer than 6 h, nanoparticles larger than 20 nm were not obtained. 

**Figure 5:**
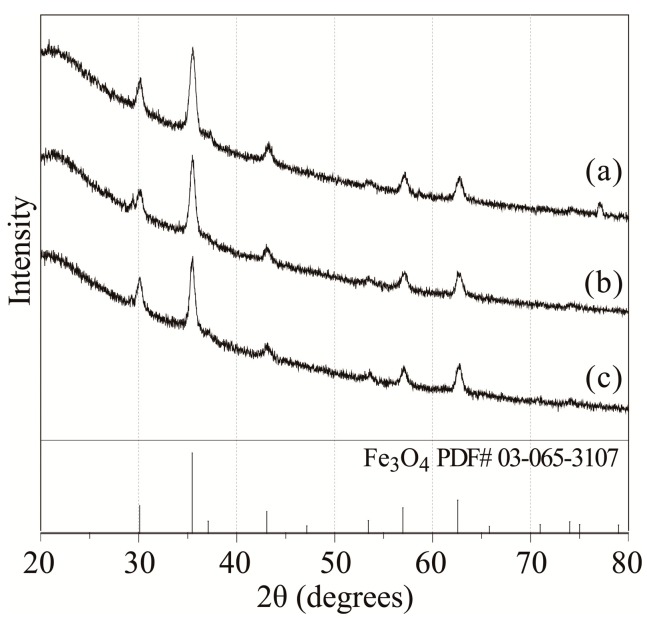
XRD patterns of (**a**) 6.6 ± 1.0 nm (1 h); (**b**) 13.0 ± 0.9 nm (3 h); and (**c**) 19.5 ± 1.7 nm (6 h) Fe_3_O_4_ nanoparticles.

**Figure 6:**
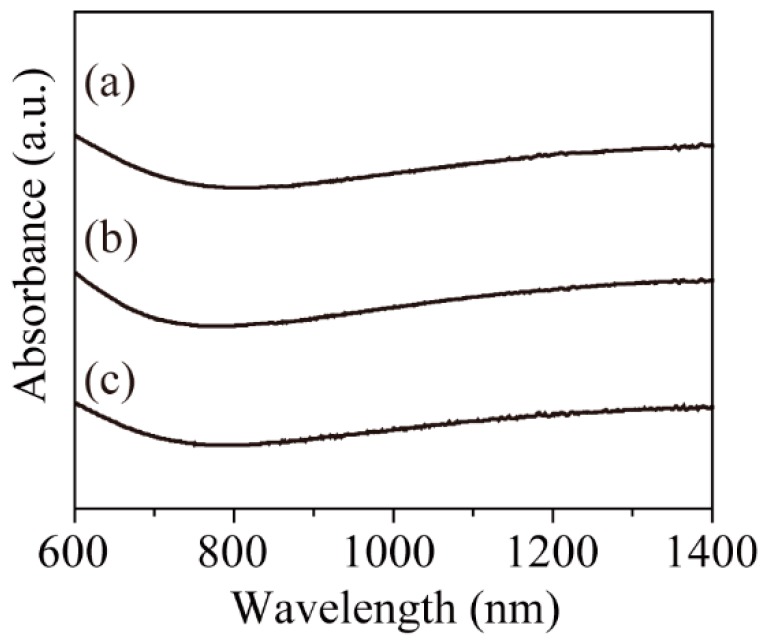
Vis-NIR spectra of (**a**) 6.6 ± 1.0 nm (1 h); (**b**) 13.0 ± 0.9 nm (3 h); and (**c**) 19.5 ± 1.7 nm (6 h) Fe_3_O_4_ nanoparticles.

Figure 5 shows the XRD patterns of the Fe_3_O_4_ nanoparticles as a function of reaction time. The patterns corresponding to the Fe_3_O_4_ phase were clearly observed in all nanoparticles. A slight decrease in peak width was observed with extended reaction time. This phenomenon depended on the particle growth and corresponded well with the TEM results. In addition, the absorbance in the NIR region derived from Fe^3+^–Fe^2+^ IVCT was observed in all nanoparticles (Figure 6). As such, a portion of the Fe^3+^ ions were reduced to Fe^2+^ and the coexistence of Fe^3+^ and Fe^2+^ ions in the nanoparticles was maintained regardless of reaction time. The particle size increased and the Fe_3_O_4_ phase was maintained with longer reaction times.

## 3. Experimental Section

### 3.1. Apparatus and Reagents

Iron(III) acetylacetonate was purchased from Kanto Chemical (Tokyo, Japan). 1,2-hexadecanediol and oleylamine (purity > 70%) were purchased from Sigma-Aldrich (St. Louis, MO, USA). Oleic acid (purity > 85%) was purchased from Tokyo Chemical Industry (Tokyo, Japan). All the reagents except oleylamine were used as received. Oleylamine was used after distillation under reduced pressure. The sizes and shapes of the resulting nanoparticles were observed by TEM (Hitachi H-7650, Tokyo, Japan, operated at 100 kV). The crystal structures were identified by XRD (Rigaku Ultima IV, Tokyo, Japan, operated at 40 kV-40 mA, Cu source). The nature of the Fe ions was determined by the absorbance in the Vis-NIR region of the sample using a Hitachi U-4100 instrument.

### 3.2. Synthesis of Fe_3_O_4_ Nanoparticles

Fe_3_O_4_ nanoparticles were prepared by the following synthetic procedure, which was based on our previous study [29]: the reaction was carried out in the 100 mL three-necked round-bottom flask equipped with a condenser and a thermometer. The heating was carried out by a heating mantle. Iron acetylacetonate (III) (1 mmol) and 1,2-hexadecanediol (3.0 mmol) as Fe^3+^ reducing agent were added into a mixture of oleic acid (15 mmol) and distilled oleylamine (15 mmol). The solution was maintained at 130 °C for 30 min with vigorous stirring under a reduced atmosphere (*ca.* 200 Pa) for dissolution and removal of impurities such as water molecules and organic molecules with low boiling temperatures. In this phase, the solution color was dark brown. Then, the solution was heated to reaction temperatures of 200 °C, 250 °C, 280 °C, and 300 °C for 1 h, 3 h, and 6 h under a nitrogen atmosphere (1 atm.). The solution color changed to black. Finally, the solution was left to cool to room temperature by remove of the heat source. When the solution becomes hard or loses fluidity after cooling to room temperature, the resulting solidified solution was dissolved by adding 10 mL *n*-hexane before the following precipitation process. The iron oxide nanoparticles were precipitated by the addition of ethanol (70~80 mL) and were subsequently subjected to centrifugation (3000 *g*, 10 min). The precipitated nanoparticles were redispersed into *n*-hexane.

## 4. Conclusions

In this study, the growth of Fe_3_O_4_ nanoparticles in excess ligands was investigated. An excess ratio of ligands to metal ions can suppress nucleation via complexation with iron ions and slowly promote particle growth. The particle sizes depended strongly on the reaction temperature due to its effect on the activity of the iron complexes, in addition to the reaction time due to the allowed growth time. By changing the reaction temperature and time, monodisperse Fe_3_O_4_ nanoparticles with tunable sizes from 5.3 nm to 20.4 nm were successfully prepared.
